# The Novel Role of PPAR Alpha in the Brain: Promising Target in Therapy of Alzheimer’s Disease and Other Neurodegenerative Disorders

**DOI:** 10.1007/s11064-020-02993-5

**Published:** 2020-03-13

**Authors:** Sylwia Wójtowicz, Anna K. Strosznajder, Mieszko Jeżyna, Joanna B. Strosznajder

**Affiliations:** 1 Department of Cellular Signaling, Mossakowski Medical Research Centre Polish Academy of Sciences, 5 Pawińskiego st., 02-106 Warsaw, Poland; 2 Faculty of Medicine, Medical University of Bialystok, 1 Kilinskiego st., 15-089 Białystok, Poland

**Keywords:** PPAR-α, Glutamatergic signaling, App/aβ metabolism, Mitochondria function, Neurodegeneration, Neuroprotection

## Abstract

Peroxisome proliferator activated receptor alpha (PPAR-α) belongs to the family of ligand-regulated nuclear receptors (PPARs). These receptors after heterodimerization with retinoid X receptor (RXR) bind in promotor of target genes to PPAR response elements (PPREs) and act as a potent transcription factors. PPAR-α and other receptors from this family, such as PPAR-β/δ and PPAR-γ are expressed in the brain and other organs and play a significant role in oxidative stress, energy homeostasis, mitochondrial fatty acids metabolism and inflammation. PPAR-α takes part in regulation of genes coding proteins that are involved in glutamate homeostasis and cholinergic/dopaminergic signaling in the brain. Moreover, PPAR-α regulates expression of genes coding enzymes engaged in amyloid precursor protein (APP) metabolism. It activates gene coding of α secretase, which is responsible for non-amyloidogenic pathway of APP degradation. It also down regulates β secretase (BACE-1), the main enzyme responsible for amyloid beta (Aβ) peptide release in Alzheimer Diseases (AD). In AD brain expression of genes of PPAR-α and PPAR-γ coactivator-1 alpha (PGC-1α) is significantly decreased. PPARs are altered not only in AD but in other neurodegenerative/neurodevelopmental and psychiatric disorder. PPAR-α downregulation may decrease anti-oxidative and anti-inflammatory processes and could be responsible for the alteration of fatty acid transport, lipid metabolism and disturbances of mitochondria function in the brain of AD patients. Specific activators of PPAR-α may be important for improvement of brain cells metabolism and cognitive function in neurodegenerative and neurodevelopmental disorders.

## Introduction

Peroxisome proliferators activated receptors (PPARs) are family of ligand-regulated nuclear receptors that include PPAR-α, PPAR-β/δ and PPAR-γ. These receptors are encoded by distinct genes: PPAR-α (NR1C1), PPAR-β/δ (NUC1 or NR1C2) and PPAR-γ (NR1C3) located on chromosome 15, 17, and 6 in the mouse and on chromosome 22, 6, 3 in humans. The PPAR-γ gene alternative promotors are responsible for three isoforms (γ1, γ2, γ3) These PPARs genes encode proteins of 468, 441, 475 and 505 amino acids with 49–56 kDa [1, 2].

**Fig. 1:**
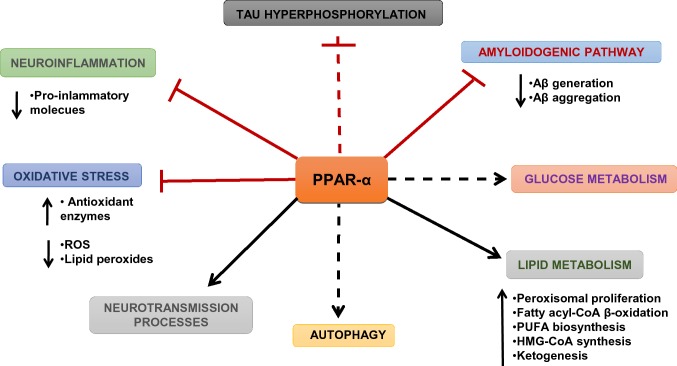
The role of PPAR-α in the brain (according to D’Orio et al. [136] with some modyfication)

PPARs regulate transcriptions through a complex mechanism as described in review by Berger and Moller [3] and Nierenberg et al. [4]. The first PPAR currently known as PPAR-α was discovered in 1990 [5]. PPARs after hetero-dimerization with RXR bind to the specific promotor region of target genes described as PPAR response elements (PPREs) and act as transcription factor(s) [4]. PPARs coactivators, such as PPAR-γ coactivator-1 alpha (PGC1-α), exert significant role in transcription of genes through interaction not only with PPARs but also with other nuclear receptors as estrogen receptors (ERs) and nuclear respiratory factors 1 and 2 (NRF1, NRF2) [6]. These receptors play a significant role in the regulation of transcription, energy and lipid metabolism and also in thermogenesis. PPAR-α regulates mitochondria metabolism, including fatty acids β oxidation pathway, energy processes, glucose metabolism, redox state and glutamatergic, cholinergic/dopaminergic neurotransmission. Additionally, PPAR-α is engaged in metabolism of amyloid beta precursor protein (APP) in the brain and directly or indirectly through Aβ it may also influence Tau protein phosphorylation. (Fig. 1) PPAR-β/δ regulates differentiation of cells, lipid metabolism and myelination processes in central nervous system (CNS) [7]. PPAR-γ and its coactivator PGC-1α play an important role in cell differentiation and mitochondria biogenesis in neurodegeneration and neuroinflammation [8–10].

The purpose of this review is to describe the role of PPAR-α in the brain in neurodegenerative and psychiatric disorders and demonstrate that this receptor could be a promising target in novel therapeutic strategies.

## PPAR-α and Its Role in Neurotransmission in the Brain

The data of Warden et al. [11] were the first to describe PPARs distribution in the brain and defined their cell type profiles in adult mouse and in human brain specific regions, which are implicated in neurodegenerative diseases. The usage of double immunofluorescence showed that only PPAR-α co-localizes with all cell types in adult mouse and adult human brain. Furthermore, the authors pointed out, that after lipopolysaccharide (LPS) injection evoking strong neuro-immune response, the significant neuronal localization of all PPARs was found with very weak co-localization of PPAR-γ in microglia.

Moreover, Roy et al. [12] determined the distribution of PPAR-α in different regions of hippocampus and observed that PPAR-α protein was localized in CA1, CA2, and CA3 and in dentate gyrus (DG) of mice brain. It was found that PPAR-α controls calcium influx and the expression of several genes coding hippocampal proteins involved in regulation of synaptic plasticity. PPAR-α is engaged in expression of *N*-methyl-d-aspartate (NMDA) receptor subunit NR2A and NR2B genes [13] and 2-amino-3(3-hydroxy-5-methyl-isoxasol-4-yl) propanoic acid AMPA receptor—associated subunit GluR1 [14], and also AMPA-receptor associated activity-related cytoskeleton proteins [15]. All these mentioned genes are related to synaptic plasticity and are regulated by PPAR-α via cyclic AMP response element binding protein (CREB). According to data of Roy et al. [12], PPAR α- null mice are deficient in CREB and memories associated proteins and have decreased spatial learning and memory.

**Fig. 2:**
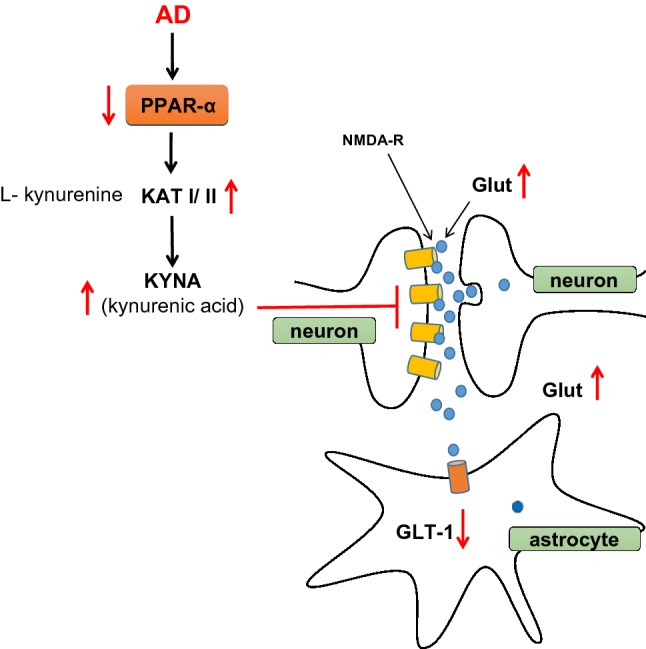
PPAR-α and it involvement in glutaminergic neurotrasmission and in glutamate homeostasis

The further study demonstrated that PPAR-α and its ligands are involved in regulation of glutamatergic and cholinergic mediated dopaminergic transmission in the brain [16–21]. The PPAR-α signaling may lead to alteration of genes transcription of enzymes that are engaged in metabolism of endogenous antagonists of glutamatergic receptors and may promote glutamate transporter-1 (GLT-1) endocytosis in astrocytes as it was showed by Huang et al. [16]. Astrocytes maintain glutamate homeostasis in CNS by glutamate uptake mediated by aspartate transporter/excitatory amino acid transporter 1 (GLAST/EAAT1) and glutamate transporter-1/EAAT2 (GLT-1/EAAT2). These two are the most important glutamate transporters in astrocytes, which remove glutamate at the synapses [22]. GLT-1 is responsible for 90% of forebrain uptake of glutamate in adult CNS and in consequence for glutamate homeostasis in the brain [23]. The imbalance of glutamate homeostasis at glutamatergic synapses could appear when astrocytic GLT-1 expression and function is decreased. It is suggested by Huang et al. [16] that agonist of PPAR-α receptor increases GLT-1 endocytosis in astrocytes through protein kinase C (PKC) signaling pathway. In AD brain the expression and function of PPAR-α is downregulated and may exert the influence on GLT-1 function (Fig. 2). Some other factors may disturb the glutamate homeostasis in AD. For example, alterations of some processes that are engaged in removal of glutamate from synaptic cleft, which can overstimulate postsynaptic glutamate receptor. In the pathological conditions glutamate transporter splice variant AEET2 may reduce glutamate uptake [24]. Moreover, Lauderback et al. [25] indicated that GLT1 could be altered by lipid peroxidation product 4-hydroxy-2-nonenal enhanced by Aβ_42_. Li et al. [26] suggested several years ago, that abnormal APP expression in AD may be responsible for GLT1 downregulation. PPAR-α agonists (GW7647 and WY14, 643) and treatment with endogenous agonist, palmitic acid (PA) significantly modulate the level of GLT-1 in astrocytes, with no changes in their morphology [16].

PPAR-α agonist, fibrate exerts neuroprotective effect by regulating the level of kynurenic acid (KYNA) an endogenous glutamate receptor antagonist. KYNA is synthesized in the brain from l-kynurenine (metabolite of l-tryptophan) by kynurenine aminotransferases (KAT) mainly by KATII isoform. An elevated brain concentration of KYNA could be responsible for memory impairment and psychotic symptoms [27, 28]. KYNA and quinolinic acid (QUIN) are major products of tryptophan metabolism, they interact differently with *N*-methyl-d-aspartate receptor (NMDA). KYNA is NMDA antagonist with neuroprotective activity but QUIN is agonist and neurotoxic. Both compounds influence learning and memory [29].

Data of Zakrocka et al. [17, 18], indicated that angiotensin II type 1 receptor blockers and agonist of PPAR-α, gemfibrozil, decrease KYNA synthesis in the brain cortex through the inhibitory effect on KATII. Zakrocka also suggested, that in the exact same way PPAR-α may exert beneficial effect in memory and in psychotic disorders. Recently several analogs of KYNA for the treatment of AD were tested and suggested for therapeutic approaches [30]. These all data suggest, that PPAR-α may play an important role in the regulation of glutamate homeostasis, which is known to be crucial in mechanism of learning and memory, in adaptive responses in neuroplasticity and in the course/outcome of several neurological diseases [31–33]. Pierrot et al. [34] reported that PPAR-α is involved in the improvement of hippocampal synaptic plasticity under RXR stimulation in experimental Tg mice model with cognitive impairment. Activation of PPAR-α receptor with specific receptor agonist enhanced transcription of GluA1 subunits of the alpha—amino-3-hydroxy-5-methyl-4-isoxazolepropionic acid (AMPA), receptor which leads to an AMPA response and better synaptic plasticity upon RXR activation. PPAR-α knockdown in the hippocampus of AD Tg cognition-impaired mice eliminated the beneficial effect of RXR activation on synaptic plasticity exclusively in males. The significant role of GluA1 subunit in long term potentiation (LTP) and in cognition was demonstrated by Schmitt et al. [35]. However, Pierrot et al. [34] shows, that LTP improvement in a Tg mouse model of AD upon RXR stimulation is correlated with up-regulation of GluA1 expression and that PPAR-α plays a crucial role in this event in a sex specific manner. The two times higher PPAR-α expression in males versus females could be responsible for the improvement of synaptic plasticity in male cognition-impaired mice by pemafibrate. PPARs and RXR receptors activation upregulates the expression of several synaptic related genes coding proteins engaged in excitatory neurotransmission [12, 36]. Probably through these mechanism agonists of PPAR-α may have a promising effect in a mouse model of aging—dependent cognitive impairments [37, 38]. Moreover, it was previously reported that RXR activation increases dendritic complexity and branching of neurons and differentiation [39, 40] but it seems now that PPAR-α plays a key role in these processes.

PPAR-α could be also engaged in cholinergic nicotinic receptor regulation of dopaminergic transmission [19, 20]. PPAR-α have been found to decrease nicotine-induced responses of dopamine neurons. Activation of nicotinic acetylcholinergic receptors (AChR) particularly those containing β2 subunits significantly regulated activity of dopaminergic neurons. The study of Melis et al. [19] using electrophysiological, biochemical and behavioral methods showed that activation of α7-nAchR enhances the β2 subunit of nAChRs and the level of endogenous agonists of PPAR-α in the animal ventral tegmental area (VTA). Their data may indicate the significant role of nAChR/PPAR-α signaling in dopamine neurons. Summarizing it is important to underline that among all PPARs only PPAR-α is involved in regulation of neurotransmission processes in the brain and in memory function. Expression of PPAR-α and PPAR-δ has been observed to be down regulated in AD [41]. PPAR-α polymorphism may be considered as a risk factor for AD [42]. In several pathological conditions including AD transcription of PPAR-α is regulated in a different way comparing to PPAR-γ [43–46]. Recently it has been observed that PPAR-α plays a significant role in microglia activation in neuroinflammation [47] and its activation evokes cytoprotective effect of statins [10].

## The Role PPAR-α in APP/Aβ Metabolism

**Fig. 3:**
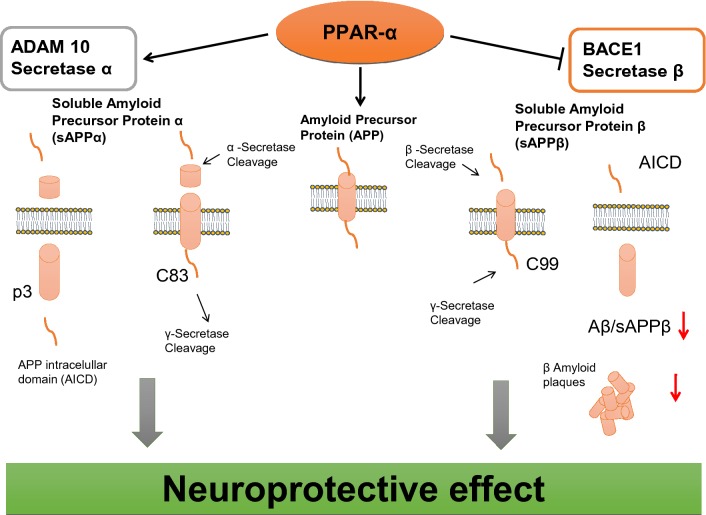
PPAR-α engagement in regulation of APP secretases in the brain

**Fig. 4:**
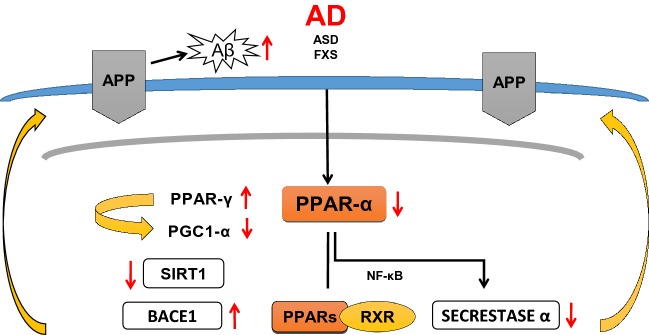
Inhibition of PPAR-α in AD brain alters APP metabolism

PPAR-α receptor plays a significant role in APP metabolism in the brain. The last data of Corbett et al. [48] indicates that under basal physiological conditions PPAR-α is involved in the degradation of APP by activation of APP α secretase leading to liberation of non-amyloidogenic peptide (p3) and soluble sAPPα with possible cytoprotective effect. The activation of this non-amyloidogenic pathway of APP metabolism may protect brain against Aβ liberation through amyloidogenic pathway in which BACE-1 plays a key role (Fig. 3). The authors show that PPAR-α agonist (gemfibrozil), enhances expression of ADAM 10 in hippocampal culture. This observation was confirmed by data on genetically modified animals (with PPAR-α coding gene knockdown). Another study reveals, that agonist of PPAR-α (WY14643) increases ADAM 10 (α secretase) expression in hippocampal neurons, however neurons lacking PPAR-α coding gene are deficient in ADAM 10 [49]. The other data of Zhang et al. [50] demonstrated, that PPAR-α agonist (GW7647) regulates amyloid β (Aβ) generation by inhibition of BACE-1 activity. Moreover, it was observed that agonist of PPAR-α receptor (GW7647) which decreased the expression of sAPPβ and the activity of BACE-1 and Aβ_1-42_ level in cell model of AD had no effect on the level of APP and Preseniline-1 (PS1). Another study of Zhang et al. [51] also showed that phosphatidylinositol 3-kinase (PI3-K) plays a significant role in PPAR signaling leading to reduction of Aβ peptides production. However, in AD alteration of PPAR-α signaling may lead to activation of APP metabolism through amyloidogenic pathway and to Aβ liberation/accumulation in the brain. It is possible that Aβ peptides liberated in excessive amount in AD may be responsible for the alteration of PPAR-α, closing the vicious circle of pathological events (Fig. 4). Some other data of Kummer et al. [52] have detected that the new PPAR agonist activates all PPARs receptors (GFT1803) decreases significantly Aβ plaques/Aβ level and microglia activation in AD mice (APP/PS1). The data suggested the role of these receptors in Aβ degradation or clearance by i.e. enhancement of insulin degrading enzyme (IDE) expression and by its activation. Moreover, pan-PPAR agonist (GFT1803) exerted positive effect on memory function affected in AD mice. Unfortunately, pioglitazone which have been investigated in clinical trial of AD patients have elicited very limited effect. Nowadays PPAR-α seems to be a promising target in AD therapeutic strategy. Anti-amyloidogenic action of PPAR-α agonists (fibrates), was observed in clinic in longitudinal treatment of patients. These people were characterized by lower Aβ_1-42_ concentration, in comparison to age related, non-treated healthy humans (control group) [53]. The last data published by Chandra et al. [54] and Chandra and Pahan [55] demonstrated that activators of PPAR-α (cinnamic acid and gemfibrozil) decrease amyloid plaque in the hippocampus and cortex in the animal model of AD (5XFAD). Moreover, they decrease neuroinflammation and thus microglia and astrocytes activation and improve spatial learning and cognition function. Activated microglia and astroglia secrete several proinflammatory cytokines and chemokines, some of which, like IL-1, TNF-α, IL-6, IL-8, and TGF-β were observed to have aberrant expression in AD patients [56].

It is known that the level of Aβ is regulated also by PPAR-γ and a number of other transcription factors which have been found to be engage in transcription of gene coding BACE-1. Among these factors some of them play more significant role, like nuclear factor kappa-light-chain-enhancer of activated B cells (NF-кB), stimulatory protein 1 (SP1), yin yang 1 transcription factor (YY1) [57]. Moreover, this study demonstrated that PGC-1α also participate in regulation of APP β secretase, BACE-1 transcription in vitro and in vivo*.* Interaction among PPAR-γ/PGC-1α and Sirtuin-1 (SIRT-1) is important in regulation of BACE-1 and is modulated by several environmental conditions including fasting [57].

These three factors interact with PPAR-RXR sites at the promotor of BACE-1 gene. The same group showed that enhancement of PGC-1α and upregulation of expression of SIRT1 leads to inhibition of BACE-1 gene expression (Fig. 4.). BACE-1 and complex of γ secretase are responsible for sequential proteolytic degradation of APP and Aβ peptide liberation, accumulation and the pathogenic protein spread of neurodegeneration in the brain of Alzheimer’ patients [58, 59]. Moreover, Qiang et al. [60] showed that SIRT 1 activates PPAR-γ directly by deacetylation. The significant role of PPAR-γ/PGC-1α in regulation of Alzheimer’s β secretase was described by Sweeney and Song [61] and Katsouri et al. [62].

The data of Qin et al. [63] demonstrated that PGC-1α gene expression is significantly decreased due to progression of clinical dementia in the Alzheimer’s brain. Moreover, it was found that PGC-1α protein content is negatively associated with Aβ_1-42_ amyloid content and AD type Aβ plaque pathology. The study on Tg2576 AD mice also indicated correlations between Aβ_1-42_ level and PGC-1α expression. PGC-1α deficiency causes behavioral changes such as anxiety, hyperactivity and hind limb clasping. Spongiform vacuolization in the striatum and other brain parts was also found [64]. Some other data suggest that PGC-1α is involved in maintenance of dendritic spines in hippocampal neurons. The authors reported that PGC-1α overexpression increases dendritic spines and enhances the molecular differentiation of synapses, whereas knockdown of PGC-1α inhibits synaptogenesis [65]. Figure 3 demonstrates the regulatory role of PPAR-α in physiological conditions on enzymes involved in APP degradation. Activation of PPAR-α induces inhibition of BACE-1 and suppression of Aβ peptides liberation/accumulation, and concomitant stimulation of α secretase, which is responsible for non amyloidogenic degradation of APP.

The higher level of Aβ observed in the brain of AD and PD patients, in the ischemic brain and the brain that was subjected to traumatic injury is postulated to be responsible for the alterations of neurotransmission processes including glutamatergic and cholinergic transmission connected with changes of PPAR-α signaling and its role in mitochondrial function.

## The Role of PPARs in Regulation of Mitochondrial Function

**Fig. 5:**
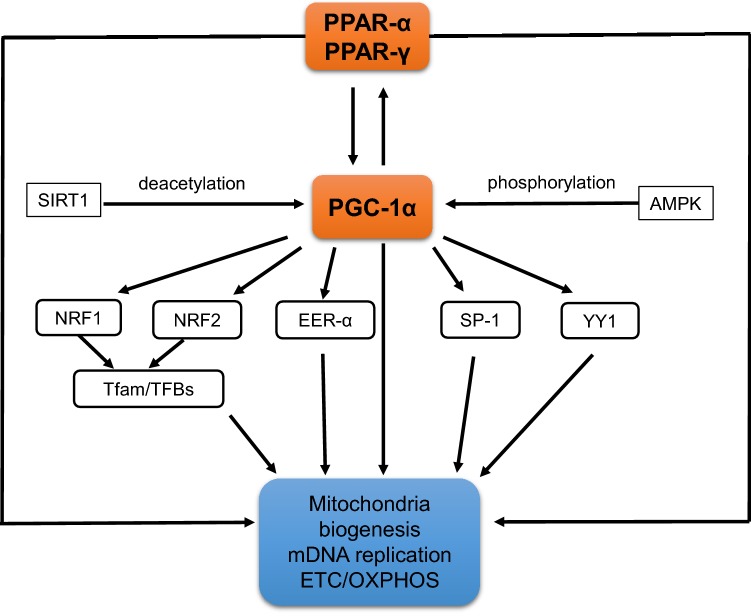
The role of PPAR-α, PPAR-γ and PGC1-α on mitochondria biogenesis and function. (according to Dominy and Puigserver [72]; Jornayvaz and Shulman [73]; Scrapulla et al. [6, 8])

Mitochondrial disturbances play a crucial role both in aging and in neurodegenerative disorders. Lamichane et al. [66] reported that PPAR-α increases genes expression encoding mitochondrial enzymes that are related to lipid metabolism, which include carnitine palmitylotransferase 1 (CPT1), medium-chain acyl-CoA dehydrogenase, acyl-CoA oxidase and fatty acyl-CoA synthase. Additionally, PPAR-α activates genes coding transport proteins of fatty acids (FA) and their derivatives to allow them enter into the β-oxidation pathway. PPAR-α induces also expression of gene that encodes protein which transfers acylcarnitine esters in exchange for free carnitine across the mitochondrial membrane [67]. Moreover, PPAR-α together with RXR activates promotor of genes coding malonylCoA dehydrogenase (MLYCD) [68, 69]. PPAR-α activates also nuclear gene encoded pyruvate dehydrogenase kinase 4 (PDK4) which is also activated by PPAR-β/δ but inhibited by PPAR-γ [70]. In addition to the PPAR-α other PPARs receptors can be also involved in activation of gene coding acyl-CoA hydrolase enzyme and ω-hydroxylase cytochrome p450 4A subfamily (CYP4A) [38, 71]. It was found that PPAR-α, PPAR-γ and its coactivator PGC-1α are very potent factors in mitochondria biogenesis through activation of mitochondrial transcription factor and several nuclear transcription factors: (TFAM-transcription factor A mitochondria, NRF1, NRF2, YY1, SP-1) (Fig. 5). Mitochondria biogenesis is regulated by different signaling pathways and transcriptional complexes that activate the formation and assembly of mitochondria [4, 6, 8, 9, 72, 73].

It is well known, that mitochondria are under significant, direct control of the nucleus. The majority of mitochondrial proteins are encoded by nuclear DNA (nDNA) similar to all nuclear transcription factors and other proteins and enzymes, including Sirtuins, poly (ADP-ribose) polymerases (PARPs) [72, 74–76]. Additionally, some of these proteins as PARP-1 are involved in DNA repair and in synthesis of Poly (ADP-ribose), (PAR) the signaling molecule that is engaged in nucleus –mitochondria crosstalk [77]. Huang et al. [16] identified PPAR-α as a substrate of PARP-1. This NAD dependent, DNA bound enzyme, is a key mediator of stress response, cells survival and death and molecular regulator of several transcription factors and other proteins. The data of Bai et al. [78] showed that PARP-1 inhibition exerts significant metabolic effect through the activation of Sirt1. Lapucci et al. [79] indicated that PARP-1 plays a key role in mitochondria homeostasis by epigenetically regulating nuclear genes involved in mtDNA repair and transcription. Nevertheless, mitochondria contain their own genome and also PARP-1 which could be involve in repair of mitochondrial DNA (mDNA) and in regulation of mitochondrial energy processes [80, 81]. It is well known that mDNA is responsible for encoding of 13 polypeptides, the subunits of respiratory Complexes I, III, IV and V as well as 2 rRNAs and 22 tRNAs [82]. The other proteins as subunits of respiratory complex II and proteins required for mDNA replication, transcription and translation and all other factors engaged in regulations of these processes are encoded by nucleus [83]. A relatively small number of nuclear factors coordinate the transcription of all respiratory proteins. Among TF are these mentioned above nuclear respiratory factors: NRF1, NRF2 then SP1, YY1 and estrogen related receptor alpha (ERR-α). Moreover, the latest data indicated presence of NAD dependent enzymes in mitochondria as PARP-1, SIRT-1 and the other Sirtuins, as SIRT-3, SIRT-4, SIRT-5, which could be involved in post translational modifications of many TF and other proteins by deacetylation, poly and mono ADP-ribosylation, sumoylation etc. [74, 75].

Numbers of components of the regulatory network proteins, such as nuclear receptor superfamily, PPARs and also DNA bound PARPs and nucleus located histone deacetylases type III, Sirtuins (SIRT-1 and SIRT-6) are implicated in mitochondria dynamics and function and may play a significant role in onset and progression of neurodegenerative diseases [75]. It is now commonly presumed, that inflammation is significantly involved in genesis and progression of most of neurological and metabolic diseases. It seems that PPAR-α, PGC-1α and the other family of nuclear receptors could be promising targets in therapeutic strategy [84].

Each of nuclear receptors PPARs has specific functions as it was described above. PPAR-α regulates oxidative stress, energy homeostasis, mitochondrial fatty acids metabolism and it may also influence excitatory glutamatergic neurotransmission and probably also cholinergic/dopaminergic signaling in brain. PPAR-β/δ and PPAR-γ regulates mainly lipids metabolism, but their specific role in the brain is yet not fully elucidated [85, 86]. There are some data demonstrated the role of PPAR-β in myelination processes in CNS [87].

PGC-1α through interaction with several nuclear receptors including PPARs can be involved in transcription regulation of genes that are related to neuro-inflammation, neuro-apoptosis and anti-oxidative defense. Moreover, PGC-1α may play a significant role in the regulation of expression of genes that are coding proteins engaged in mitochondria biogenesis, DNA level and mitochondria function [73]. PGC-1α may regulate mitochondria oxidative metabolism by activating genes that are involved in the tricarboxylic acid cycle (TCA), oxidative phosphorylation, β-oxidation of fatty acids [88]. PPARs are involved in activation of PGC-1α expression, which subsequently plays an important role as coactivator of these receptors and regulator of PPARs transcription. These relationships may lead to some type of molecular vicious circle. PGC-1α is activated by NAD regulated enzymes, by SIRT-1 mediated deacetylation, or by phosphorylation mediated by 5′ adenosine monophosphate-activated protein kinase (AMPK) and other kinases. p38 [64]. It was observed that gene expression and protein level of PGC-1α dependently on pathological conditions of CNS could be differently affected. There are several data reporting down regulation of PGC-1α expression and activity in AD and in the other pathological conditions as Huntington disease (HD) brain ischemia and inflammation [52, 57].

Patients with Huntington’s disease (HD) had a decreased level of PGC-1α [89, 90] which may suggest that PGC-1α is crucial for striatal cells function. In Parkinson Disease (PD) mouse model, PGC-1α deficiency is suggested to be involved in degeneration of dopaminergic neurons in the *substantia nigra*. The protective role of PGC-1α was demonstrated in Amyotrophic Lateral Sclerosis (ALS) mice and ALS patients by Thau et al. [91].

Recently it is also suggested, that PGC-1α plays a significant role in brain ischemic encephalopathy, hepatic encephalopathy, brain alterations in diabetes type I and diabetes type II and in neurodegenerative processes evoked by acute and chronic brain injury which may lead to traumatic encephalopathy [64, 92]. PGC-1α synthesis is regulated by changes in environmental conditions and it is believed that this factor can link signal from the environment to gene expression in the brain. PGC-1α is expressed in all brain tissue and in most brain regions and plays a key role in the oxidative stress response [92, 93]. Mechanism of neuroprotection mediated by PGC-1α signaling pathway is very complex as it was demonstrated recently by Lv et al. [92]. Summarizing PGC-1α has an influence on cellular metabolism and inflammatory response through regulation of mitochondrial biogenesis, oxidative metabolism, fatty acid oxidation and gluconeogenesis in mechanism dependent on PPARs function. PGC-1α is also able to regulate not only PPARs but also other nuclear receptors, such as estrogen receptors. Despite the growing number of reports on the role of PGC-1α in neuroprotection, the mechanism of PGC-1α in regulation of gene expression and mitochondria dynamic and function in different pathological conditions is not fully elucidated.

## PPAR-α Natural and Synthetic Agonists: from Experiments to Clinical Trials

PPAR-α regulates oxidative stress, energy homeostasis, mitochondrial fatty acids metabolism and is only one among all PPARs which influence excitatory glutamatergic neurotransmission and also cholinergic/dopaminergic signaling in the brain. Expression of PPAR-α but also other PARPs is significant in prefrontal cortex, nucleus accumbens and amygdala in the brain and significantly higher in neuronal cells versus glia cells [11].

The specific role of PPAR-β/δ and PPAR-γ in the brain is yet not fully elucidated [85, 86]. There are some data demonstrating the involvement of PPAR-β in myelination processes in CNS [87]. On the basis of data, it is now expected that activation of PPAR-α should be most promising in therapeutic strategy of several neurodegenerative and metabolic disorders.

The data of Esmaeili et al. [84] demonstrated, that preferential activation of PPAR-α by fenofibrate reduces neuroinflammation and blocks neurodegeneration in mice model of ALS. In animals treated with fenofibrate, the mRNA analysis indicated significant effect of this drug on transcription of genes that encode proteins engaged in anti-inflammatory and anti-oxidative defense. It also down regulates expression of genes that are involved in neuro-inflammation. In PD animals model treated with agonist of PPAR-α, mitochondria were preserved and treated mice exhibited enhanced motor performance. Fenofibrate treatment significantly delayed progression of disease.

In other experimental animal model, that was part of the study on PD, Uppalapati et al. [94] was also observed neuroprotective effect of PPAR-α agonist, fenofibrate/fenofibric acid. This compound exerted significant ameliorating effect on cognitive impairment. About 40% of PD patients are affected with cognitive impairment and dementia, which are about 6 times more frequent in PD patient comparing to healthy, age related control people. Several previous studies indicated promising effects of PPAR agonists in PD [95–98]. Previously also Carta et al. [99] reported ameliorating effect of rosiglitazone which decreased PPAR-γ level and inhibited TNFα production in progressive PD mice model. Another randomized trial of pioglitazone did not confirm the ameliorating effect of this drug in PD [4]. A significant effect of PPAR agonists was observed in treatments of depressive states which often occur also in prodromal stage of PD being therefore considered as a very important early symptom of PD. Further investigations are necessary to evaluate the role of PPARs in PD.

The study of the last decade indicated the important role of PPAR-α in cognition and emotions [100]. Their data underline the relationship between function of PPAR-α, its endogenous agonists and the level of neurosteroids in stress conditions. PPAR-α is high in these brain regions which participate in the regulation of emotions and stress response.

The highest expression of PPAR-α was found in prefrontal cortex, basal ganglia, amygdala, and in thalamic nuclei, but its expression is way lower in hippocampus [11]. The data of Nisbett and Pinna [100] demonstrated that PPAR-α activation mediate and modulate stress response observed also by Hillard [101]. It was proposed that in the mechanism of PPAR-α action on emotion and stress key role may play neurosteroids such as allopregnanolone/prognanolone [102]. Basing on the correlation between PPAR-α stimulation and allopregnanolone biosynthesis the novel biomarker axis and therapeutic strategy is proposed for emotional alterations observed in posttraumatic stress disorders (PTSD) and other mood disorders [100]. Agonist of PPAR-α receptor may exert neuroprotective effect also by enhancing the level of brain derived neurotrophic factor (BDNF). Jiang et al. [103] reported antidepressant-like effect of fenofibrate in mice via PPAR-α mediated BDNF signaling pathway in hippocampus. The antidepressive—like effect of fenofibrate in the mice model was blocked by PPAR-α inhibitor and lower hippocampal BDNF signaling pathway. Moreover, the same group [103] postulated that the antidepressant-like effect of fenofibrate do not require the cannabinoid system. Several studies supported the crucial role of BDNF signaling alterations in pathophysiology of depression [104, 105]. Previously it was observed that pioglitazone significantly improves depressive symptoms and significantly reduces the level of pro-inflammatory cytokines in bipolar disorder as reported by Kemp et al. [106] and Zeinoddini et al. [107]. Improvements of depression severity were also observed in unipolar depression by Kemp et al. [108, 109] as well as by Lin et al. [110] in double–blind placebo—controlled trial.

On the basis of actual data PPAR-α and PPAR-γ receptor agonist and also PGC-1α are further proposed as a promising target in therapy of metabolic and inflammatory disorders as well as other neurological and neuropsychiatric diseases. In several neurodegenerative diseases PGC-1α expression is downregulated together with its target genes that code NRF1 and TFAM. This data suggested that signaling pathway mediated by these proteins is crucial in neurodegeneration/neuroprotection. PGC-1α and PPARs are important in adaptive responses of neuronal mitochondria to bioenergetics challenges [32]. Moreover, it is important to underline that PGC-1α negatively regulates extra synaptic glutamatergic receptor (NMDAR) activity and excitotoxicity [111]. The studies of Vallee and Lecarpentier [49] on AD described that PPAR-γ agonists diminish learning and memory deficit in AD patients.

Recently few clinical trials indicated and suggested that PGC-1α through its interaction with PPARs could have promising effects in a therapy of mood alterations, particularly in the treatment of depressed states. Sepanjnia et al. [112] reported that pioglitazone therapy improved the mood in depressive episodes. The mechanism of anti-depressive action of the pioglitazone should be better elucidated. Changes in the level of several endogenous ligands of PPAR-γ including fatty acids and prostaglandins may exert additional effect on the signaling pathway.

The endogenous compounds are weak agonists and a role of its interactions with PPARs receptors in physiological and pathological conditions is not fully understood. Several compounds were synthesized, among them thiazolidinediones (also called glitazones), which are strong agonists. These compounds were studied in many diseases and were used in clinical treatment of obesity and other metabolic disorders. Up till now rosiglitazone (BRL-49653) and pioglitazone were recommended for diabetic type II treatment. Most of compounds from this group of drugs including rosiglitazone were removed from the USA market by FDA due to their dangerous side effects like increase the risk of myocardial infraction [113].

Recent study indicated that PGC-1α and agonists of pan—PPARs may play an important role in autism spectrum disorder and other neuropsychiatric alterations [114]. It was indicated that agonists of PPAR-α and also all PPARs could be involved in the bipolar disorders and in depression [4]. They also have an influence on behavioral repetition and cognitive flexibility in mice [115]. Additionally, the protective effect of PPAR-α agonist was showed in rat model of oral dyskinesia and in different types of encephalopathy [116, 117]. Moreover, PPARs agonists may also affect alcohol consumption behavior [118]. There are several data indicating, that neuroprotective effect of PPAR-α mediate the action of resveratrol against stroke which is similar as the effect of fenofibrate [119]. PPAR-α receptor stimulation induces synthesis of allopregnanolone in astrocytes and this hormone might be involved in neuroprotective mechanism [120]. Therapeutic effect of PPAR-α on neuronal death and microvascular impairment was described by Moran and Ma [121]. PPAR-α and other receptors from this family play significant role in regulation of oxidative stress and inflammatory reactions. It is now accepted, that oxidative stress is considered to be a crucial factor in pathogenesis and pathomechanism of neurodegenerative and neurodevelopmental/psychiatric disorders. Recently mitochondrial oxidative stress is believed to be associated with autism spectrum disorders (ASD), which is frequently diagnosed among children. This disorder is induced by genetic/chromosomal abnormalities and environmental factors, which could include prenatal exposure to stress, altered hormone levels or different teratogenic compounds. This multi-factors disorder, ASD, is now observed in as many as 1 in 80 children, or even 1 in 41 children and the proper therapy has not been yet discovered. It was observed and suggested, that alteration of presynaptic proteins, including APP, that till now were mostly investigated in relation to AD, occurs also in ASD. APP is known to play a very important role in synaptogenesis, in neurite outgrowth, in integrity of brain development, memory formation and in neuronal plasticity. Unfortunately, its expression and degradation is significantly affected in the brain of ASD. Amadei et al. [122] reported, that β amyloid plaques occur also in the brain of children with ASD. Additionally, elevated level of soluble sAPPα was detected earlier in the blood of autistic children. It was observed, that agonist of PPARs, pioglitazone, GFT1803 (Pan-PPAR agonist), protects brain against amyloidβ deposition and cognition impairment [52].

The two neurodevelopmental disorders—ASD and Fragile X Syndrome (FXS) are connected with altered APP metabolism and with Aβ plaque formation, which may affect learning and memory function. It is suggested, that sAPP, Aβ_1-42_, Tau protein and also ApoE allele should be considered as a promising biomarkers not only for AD but also for ASD and perhaps other neurodevelopmental disorders. Several data indicated that PPARs/PGC-1α and SIRT-1 are involved in regulation of BACE-1 transcription, the key enzyme for Aβ generation. Data of Wang et al. [57] demonstrated that BACE-1 promotor contains multiple PPAR-RXR sites. Previous results indicated, that BACE-1 expression is regulated significantly by several transcription factors in AD and in ASD and inflammation by NF-кB and PPAR [123, 124].

The pathogenesis/pathomechanism of neurodegenerative diseases is not yet elucidated and therapy is not successful. Several pharmacological compounds including PPARs agonists were applied in the treatment of patients with ASD, AD, PD, ASL, however many side effects of PPARs agonists were observed and described recently [4].

Nowadays there are novel research directions exploring the potential of PPAR-α agonists including natural and synthetic compounds as demonstrated in Tables 1 and 2 respectively. Moreover, some studies are directed to find novel promising dual and pan PARPs agonists for treatments of inflammatory and neurodegenerative disorders. The pan PPAR agonist, bezafibrate is used with success for over 25 years in the treatment of patients with metabolic syndrome, DM-TII, in antycholesterolemia strategy and in protection against myocardial disorders [125]. Bezafibrates have positive effects on mitochondria function and on behavior in PD and HD [64, 126]. Their action trough three receptors types may lead to the elimination of negative influences of PPAR-γ on cardiovascular system, metabolic rate and body weight. Unfortunately, a lot of clinical trials with agonists of PPARs in neurodegenerative diseases are controversial, due to the fact, that there are studies suggesting their ineffectiveness. Some other last data indicate promising, positive effect of PPARs agonists [127–135]. Overall it seems that personalized gene-related therapy or personalized combination of specific pharmacological compounds should be more efficient. The further studies on pharmacologically active compounds that target PGC-1α and PPARs are necessary.

**Table 1:** Agonists of PPAR-α—natural and synthetic (according to data published by Adedapo et al. [127]; Singh et al. [128]; Rigano et al. 2017 [129]; and Contreas et al. [130], Fournier et al. [2])

PPAR-α agonist			
Natural			Synthetic
Endogenous	Exogenous		
Oleoylethanolamine (**OEA**)	Monoterpenes	Linalool	2-(4-Chlorophenoxy)-2-methyl-propanoic acid, ethyl ester **Clofibrate**
Palmitoylethanolamide (**PEA**)	Sesqui terpenes	*Trans* caryophyllene	2-[4-[2-[(4-Chlorobenzoyl)amino]ethyl]phenoxy]-2-methylpropanoic acid Bezafibrate
8-hydroxyeicosatetraenoic acid (**8 (s) HETE**)		Farnesol	Propan-2-yl 2-[4-(4-chlorobenzoyl)phenoxy]-2-methylpropanoate **Fenofibrate**
8-hydroxyeicosapentaenoic acid (**8 (s) HEPE**)	Diterpenes	Phytol	(5-(2,5-Dimethylphenoxy)-2,2-dimethylpentanoic acid) **Gemfibrozil**
Arachidonic acid (**ARA**) (**C20:4**)	Triterpenes	Oleanolic acid	
	Anthraquones	Norathyriol	(2-(4-(2-(1-Cyclohexanebutyl)-3-cyclohexylureido)ethyl)phenylthio)-2-methylpropionic acid) **GW 7647**
Leukotriene B4	Pnenylopro panoids	Rosmarinic acid	(2-[[4-[2-[[[(2,4-Difluorophenyl)amino]carbonyl]heptylamino]ethyl]phenyl]thio]-2-methyl-propanoic acid) **GW 9578**
Eicosapentaenoic acid (**EPA**) (**C20:5**)		Verbascoside	2-Methyl-2-[4-[3-[1-[(4-methylphenyl)methyl]-5-oxo-2H-1,2,4-triazol-3-yl]propyl]phenoxy]propanoic acid **LY 518674**
Linoleic acid (**LA**) (**C18:2**)	Coumarins	Coumarin	2,2-Dichloro-12-(4-chlorophenyl)dodecanoic acid **K 111**
Palmitic acid (**PA**) (**C16:0**)	Lignans	Sesamin	(S)-3-[3-(1-carboxy-1-methyl-ethoxy)-phenyl]-piperidine-1-carboxylic acid **CP 900691**
Stearic acid (**SA**) (**C18:0**)	Polyphenoles	Pterostilbene	4-Chloro-6-(2,3-xylidino)-2-pyrimidinylthioacetic acid, Pirinixic acid **WY 14643**
	Flavonoides	Hispidulin	
		Wagonin	2-Methyl-c-5-[4-[5-methyl-2-(4-methylphenyl)-4-oxazolyl] butyl]-1,3-dioxane-r-2-carboxylic acid (NS 220)
		Epigallocatechin	
	Isoflavonoids	Genistein	
		Daidzein	
		Biochanin A	
		Formononetin	
		Tectoridin	
	Bioflavonoids	Bilobetin	
	Alkaloids	Picrasidine C	
		Berberine	
		Oxymatrine	
		Capsaicin

**Table 2:** Effect of synthetic PPAR-α agonists in clinical trials and experimental models of neurodegenerative/psychiatric disorders

Clinical study OF PPAR-α agonists				
Drug name	Disease	Main effect	Clinical trial	References/clinical trial identifier
Gemfibrozil	Alzheimer’s disease	Downregulates of BACE1 expression	II phase pending	NCT02045056
Fenofibrate	Drug-resistant Nocturnal frontal lobe epilepsy	Reduces of seizure frequency Effects on motor-behavioral seizures	II phase	[131]
Gemfibrozil	Alcoholism	Influences drinks per drinking day and percent days abstinent	II phase completed	NCT02158273
Fenofibrate		Reduces craving to drink and drinks per week	II phase terminated	NCT03539432
Bezafibrate	Bipolar disorder	Positive change in Montgomery-Åsberg Depression Rating Scale	I phase ongoing	NCT02481245

Study in experimental models of PPAR-α agonists				
Drug name	Disease	Main effect		References
Gemfibrozil	Alzheimer’s disease	Stimulates ADAM10 Reduces Aβ production		[48]
		Decreases Aβ plaque accumulation Improves learning memory		[55]
		Decreases Aβ accumulation and reverses memory deficits and anxiety symptoms		[132]
WY-14643		Decreases tau protein and inflammation markers Increases ability in Moris water test		[37]
GW7647		Regulates APP amyloidogenic processing Decreases the expression of sAPPβ and ability of BACE1 Reduces Aβ release and Aβ production		[50]
Fenofibrate		Regulates oxidative stress accumulation		[133]
Fenofibrate	Parkinson’s disease	Protects against the damaging effect of MPTP in a rat model Decreases inflammation		[94, 134]
	Cerebral injury	Reduces suspectibility to stroke in apolipoprotein E-deficient animals Decreases cerebral infarct volume		[135]

## Summarizing

This review focused on the role of PPAR-α and it interaction with PPAR-γ/PGC-1α in the brain during neurodegenerative and neuropsychiatric disorders. PPAR-α is involved in regulation of glutamate homeostasis and in some aspects also in cholinergic and dopaminergic signaling. Only PPAR-α receptor plays a significant role in transcription of genes coding proteins that are engaged in neurotransmission processes in the brain. However, further studies are necessary to understand the role of PPAR-α in glutamatergic and other signaling pathways in physiological conditions and in AD or other neurodegenerative/neurodevelopmental diseases.

The recent data demonstrated, that PPAR-α is engaged in APP metabolism through amyloidogenic pathway. PPAR-α activates α secretase and inhibits BACE-1, the key enzyme responsible for Aβ peptides liberation. However, nothing is known on its role on Aβ peptides transport and on mechanism of it degradation in AD.

In AD, PPAR-α opposite to PPAR-γ is down regulated similar to PGC-1α, however the mechanism of the PPAR-α signaling alterations in AD hasn’t been fully elucidated yet.

In mitochondria PPAR-α is involved in regulation of fatty acid and energy metabolism. However, its significance in regulation of mitochondria biogenesis, dynamics and function of electron transport complexes as well as the effect of fenofibrates, agonists of this receptor used for many years in therapy of diabetes type II, obesity and other metabolic disorders hasn’t been fully analyzed up till now. It seems now that PPAR-α could be promising target for the novel therapeutic strategy of AD and other neurodegenerative and neurodevelopmental disorders. However, the mechanism of its action in the brain should be characterized in depth to enable successful application.
